# Spectral, Magnetic and Biological Studie on Some Bivalent 3d Metal Complexes of Hydrazine Derived Schiff-Base Ligands

**DOI:** 10.1155/MBD.1997.65

**Published:** 1997

**Authors:** Zahid H. Chohan, Syed K. A. Sherazi

**Affiliations:** 1 Department of Chemistry Islamia University Bahawalpur Pakistan; 2 Department of Chemistry Meston Walk University of Aberdeen Scotland Old Aberdeen AB9 2UE UK

## Abstract

Metal(II) complexes of hydrazine derived Schiff-base ligands of the type M(L)_2_Cl_2_ where M = Co, Cu, Ni and Zn and L = L_1_ and L_2_ have been prepared and characterised by molar conductance, magnetic moment, elemental analysis and electronic, IR, H-NMR and ^13^C spectral data.The different modes of chelation of the ligands and their comparative biological properties against different bacterial species are reported.


### SPECTRAL, MAGNETIC AND BIOLOGICAL STUDIE ON SOME BIVALENT 3d METAL COMPLEXES OF HYDRAZINE DERIVED SCHIFF-BASE LIGANDS

Zahid H. Chohan* and Syed K. A. Sherazi

Department of Chemistry, Islamia University, Bahawalpur, Pakistan

### ABSTRACT
Metal(II) complexes of hydrazine derived Schiff-base ligands of the type $M(L)_2Cl_2$ where M = Co, Cu, Ni and Zn and L = $L_1$ and $L_2$ have been prepared and characterised by molar conductance, magnetic moment, elemental analysis and electronic, IR, H-NMR and $^{13}C$ spectral data.The different modes of chelation of the ligands and their comparative biological properties against different bacterial species are reported.

### INTRODUCTION
In view of the promising role[1,2] of Schiff-bases as ligands in metal coordination chemistry, we have commenced a research program[3-8] to study the ligational and biological behaviour of different Schiff-base ligands.The present work,with the same idea has been undertaken and extended to the hitherto less investigated Schiff-base ligands derived from hydrazines and their complexes with 3d metal ions.These studies might permit us to report a variety in coordination behaviour of hydrazines.

Many reports[9-12] on coordination properties of acyl and aroyl hydrazines have appeared.We have already reported[13] pyrrolyl, thienyl and furanyl derived hydrazines and their 3d metal complexes and in continuation to the same, now, wish to report the synthesis,structural studies and biological behaviour of 3d metal ions such as Co, Cu, Ni & Zn on the title ligands $L_1$ and $L_2$.

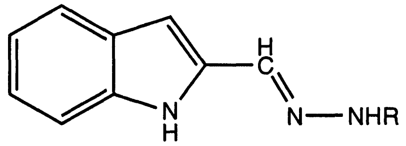

Fig .1: Structure of the Ligands ($L_1$ : R = Ph, $L_2$ : R = H)

### EXPERIMENTAL
### Material and Methods
All the chemicals used were of Analar Grade.Metal ions were used as their chloride salts.Conductance and magnetic measurements were made on a YSI model-32 conductivity bridge and Gouy balance, respectively.IR spectra were recorded on a $R_{10}$ Hitachi spectrophotometer. H-NMR spectra of the ligands in DMSO-$d_6$ were obtained on $R_{10}$ Perkin-Elmer spectrometer.13C NMR spectra of the ligands were obtained on a Brucker 250 MHz instrument.Electronic spectra were studied in DMF on a Hitachi double-beam U-2000 model spectrophotometer using glass cells of 1 cm thickness.Elemental analysis of C, H & N were determined on a Coleman automatic analyser.All melting points were taken on a Gallenkamp melting point apparatus and are uncorrected.All the complexes were analysed for their metal contents employing standard literature procedures[14] after decomposing the organic matter at first with a mixture of conc $HNO_3$ and HCl and then with conc $H_2SO_4$.Chloride was estimated as AgCl and nitrogen as microanalytically.

Antibacterial studies were carried out with the help of the Microbiology Laboratory, Department of Microbiology,Qaide Azam Medical College, Bahawalpur.These studies were done on wild pathogenic bacterial species collected from urine and blood samples of infected patients admitted in Bahawal Victoria Hospital, Bahawalpur.

### Preparation of the Ligands
#### N-3-(Indolylmethylene) phenyl hydrazine ($L_1$).
Indole-3-carboxaldehyde(0.4 g, 0.01 mol) in ethanol (15 mL) was added to an ethanolic solution (20 mL) of phenyl hydrazine (0.7 g, 0.01 mol).Then 2-3 drops of conc.$H_2SO_4$ were added and mixture refluxed for 1h. The reactant mixture on cooling gave a yellow solid product which was filtered, washed with ether and dried. It was crystallised in hot aqueous ethanol to give $L_1$ (72 %). The same method was adopted using the same molar ratio of respective reagents for the preparation of $L_2$ (75 %).

* Present address: Department of Chemistry, Meston Walk, University of Aberdeen, Old Aberdeen AB9 2UE, Scotland, U.K.

**Preparation of the Metal Complexes**

To a hot ethanolic solution (20 mL) of the ligand (0.02 mol) was added an aqueous solution (15 mL) of the respective metal(II) chloride (0.01 mol).The mixture was refluxed for 1h.The resulting mixture was cooled,filtered and reduced to nearly half its volume.It was then left overnight at room temperature which resulted in the formation of solid product.The product thus obtained was filtered, washed with ethanol (2x10 mL), then with ether (10 mL) and dried.Crystallisation in hot aqueous ethanol (50 %) gave **1** (55 %), **2** (57 %), **3** (52 %), **4** (55 %), **5** (50 %), **6** (58 %), **7** (50 %) and **8** (48 %).

### RESULTS AND DISCUSSION

The Schiff-base ligands were prepared by the same method reported earlier[5-8].The structural determination of these ligands was done with the help of their spectral and analytical data.

**Table 1** Physical, Analytical and Spectral Data of the Ligands

| Ligand/ Mol.Form. | M.P (°C) | IR($cm^{-1}$) | $^1$H-NMR(ppm) | $^{13}$C-NMR(ppm) | Cal(Found)% C H N |
|---|---|---|---|---|---|
| $L_1$ $C_{15}H_{13}N_3$ | 175-177 | 3215,3190, 3100,2920, 2775,2516, 2020,1625, 1545,1460, 1345,1211, 1135,950 | 4.64(s,1H,aromatic), 6.1(s,1H,azomethine), 8.37 (s,2H,NH), 7.45-7.48(m,3H,m,p-Ph), 8.85-8.87(m,2H,o-Ph) | 108.51($C_3$),112.29 ($C_8$),115.7(o-Ph), 120.61(p-Ph), 121.66 ($C_5$),123.48($C_6$), 124.75($C_7$),128.07 ($C_4$),129.57(m-Ph), 152.42(ipso),156.22 ($C_9$),158.10($C_2$), 165.7(azomethine) | 76.61 5.52 17.86 (77.03)(5.28)(17.57) |
| $L_2$ $C_9H_8N_3$ | 158-160 | 3215,3190, 3100,2925, 2015,1625, 1545,1135, 955 | 4.63(s,1H,aromatic), 6.7(s,1H,azomethine), 8.34(s,1H,NH), 7.18-7.29 (m,2H,aromatic), 7.45-7.71 (m,2H,aromatic), 8.85(s,2H,$NH_2$) | 108.48($C_3$),112.16 ($C_8$)121.67($C_5$), 123.47($C_5$),124.77 ($C_7$),128.1($C_4$), 156.22($C_9$),158.16 ($C_2$),165.73 (azomethine) | 68.37 5.06 26.56 (68.72)(5.11)(26.38) |

The IR spectra of the free ligands (Table 1) show some characteristic bands at 3215, 3100, 1625, 1545 and 950-955 $cm^{-1}$ assigned[15] respectively to ($-NH_2$), (-NH), (-C = N), (-C = C-) and (-N - N) frequencies. All ligands showed the absence of a strong band at 1740 $cm^{-1}$ due to carbonyl (-C = O) stretching and similarly, the appearance of a new band at 1625 $cm^{-1}$ due to azomethine(-C = N) linkage provided a strong evidence for the formation of ligands $L_1$ and $L_2$.Also $^1$H-NMR and $^{13}$C spectral data (Table 1) of the title ligands showed all the expected protons and carbons on the expected ppm values. Their CHN percentage, however, confirmed the molecular formulae and their structures. The reaction of the ligands with metal(II) salts yielded complexes (1-8) (Table 2)of composition M : L = 1 : 2. All the synthesised complexes are air/moisture stable solids.They are soluble in DMF, DMSO and $H_2O$ and partially soluble in common organic solvents e.g., chloroform,ethanol,acetone and benzene. Their melting temperatures, solubility and crystalline nature also suggested[16,17] that they are all non-polymeric. The room temperature magnetic susceptibility measurements (Table 2) on the solid complexes indicate three unpaired electrons per Co(II) ion (3.34-3.60 B.M), one unpaired electron per Cu(II) ion (1.78-2-16 B.M) and two unpaired electrons per Ni(II) ion (2.96-3.06 B.M) which strongly suggest[18-21] octahedral geometry for Co(II) and Ni(II) complexes and distorted octahedral environment for Cu(II) complexes.

The comparative studies of the IR spectra of the ligands and their metal complexes indicated that the ligands are coordinated to the metal atom possibly in three ways a) The bands at 3215 and 3100 $cm^{-1}$ attributed to(-NH2) and (-NH) modes in the spectra of the ligands suffer a negative shift indicating the involvement of this group.

b) The band in the spectra of the ligand at 1625 $cm^{-1}$ due to the azomethine (-C = N) linkage is also shifted towards lower frequency side by 5-10 $cm^{-1}$ respectively, suggesting the ligand to be coordinated to the metal atom through azomethine nitrogen.

c) The new band appearing in the spectra of the metal (II) complexes and not observed in the spectra of ligands at 515-520 $cm^{-1}$ assigned[15] to M-N mode respectively indicated that the heteroatom X (Fig 2) is also coordinated to the metal(II) ions.

The above observations gave a conclusive evidence of the coordination between metal(II) ions and the ligands possibly through NH or X, $NH_2$ and C = N (azomethine) groups.

The electronic spectra of the Co(II) complexes exhibited three bands at 9305-9515, 15675-15810 and 21275-21330 $cm^{-1}$ assigned to the transitions $^4T_{1g}(F) \rightarrow {}^4T_{2g}(F)(V_1)$ $^4T_{1g}(F) \rightarrow {}^4A_{2g}(F)(V_2)$ and $^4T_{1g}(F) \rightarrow {}^4T_{1g}(P)(V_3)$ respectively assuming octahedral coordination around the metal ion[22,23]. The Cu(II) complexes showed, similarly three bands in the region 14510-15155, 22480-22585 and 31100-31270 $cm^{-1}$.The first two bands are attributed to d-d transitions for their distorted octahedral configuration[24] while the third may be attributed to intra-ligand charge transfer.The electronic spectra of Ni(II) complexes also showed three bands in the region 10120-11605, 16062-16180 and 25210-25575 $cm^{-1}$ assignable, respectively to the transitions $^3A_{2g}(F) \rightarrow {}^3T_{2g}(F)(V_1)$, $^3A_{2g}(F) \rightarrow {}^3T_{1g}(F)(V_2)$ and $^3A_{2g}(F) \rightarrow {}^3T_{1g}(P)(V_3)$ consistent with idealised octahedral geometry[25,26].Also, Zn(II) complexes showed a charge transfer band at 29515-30115 $cm^{-1}$ and a band at 13111-13280 $cm^{-1}$ due to transitions $^2E_g \rightarrow {}^2T_{2g}$ in a distorted octahedral environment[27].
On the basis of the above observations,it is proposed that all the metal complexes show an octahedral geometry in which the two ligands behaving as tridentate accommodate themselves around the metal ion in such a way that a stable chelate ring is formed(Fig 2) attaining a stable configuration of a metal(II) complex.

**Table 2** Physical, Analytical and Spectral Data of Metal Complexes

| No/Complex | M.P (°C) | B.M ($\mu_{eff}$) | IR($cm^{-1}$) | $\lambda_{max}$($cm^{-1}$) | Cal (Found)% C H N |
|---|---|---|---|---|---|
| 1 $[Co(L_1)_2Cl_2]$ | 210-212 | 3.60 | 3210,3185,2982, 2920,2714,1621, 1545,1364,1130, 950,515 | 9515,15675, 21330 | 57.54 8.30 13.41 (57.88)(8.02)(13.89) |
| 2 $[Co(L_2)_2Cl_2]$ | 197-199 | 3.34 | 3212,3185,2980, 2922,2715,1620, 1545,955,518 | 9305,15810, 21275 | 48.90 2.71 19.00 (49.13)(2.69)(19.82) |
| 3 $[Cu(L_1)_2Cl_2]$ | 228-230 | 2.16 | 3210,3182,2985, 2920,2715,1620, 1545,1135,1025, 950,522 | 14510,22485, 31270 | 57.12 8.24 13.31 (57.44)(8.09)(12.98) |
| 4 $[Cu(L_2)_2Cl_2]$ | 217-219 | 1.78 | 3212,3185,2982, 2920,1625,1545, 950,515 | 15155,22585, 31100 | 48.40 2.68 18.80 (48.47)(2.33)(18.93) |
| 5 $[Ni(L_1)_2Cl_2]$ | 233-235 | 3.06 | 3212,3185,2980, 2920,2715,1620, 1545,1340,1135, 950,515 | 11605,16062, 25210 | 57.56 8.30 13.42 (57.55)(8.79)(13.61) |
| 6 $[Ni(L_2)_2Cl_2]$ | 221-223 | 2.96 | 3210,3182,2985, 2920,2715,1625, 1545,950,515 | 10120,16185, 25575 | 48.93 2.71 19.01 (49.37)(2.78)(18.92) |
| 7 $[Zn(L_1)_2Cl_2]$ | 215-217 | Dia | 3210,3185,2982, 2925,2715,1620, 1154,955,515 | 13280,29515 | 56.95 8.22 13.27 (57.08)(7.86)(13.33) |
| 8 $[Zn(L_2)_2Cl_2]$ | 208-210 | Dia | 3210,3185,2983, 2920,2715,1620, 1545,955,515 | 13111,30115 | 48.20 2.67 18.73 (48.33)(2.38)(19.03) |

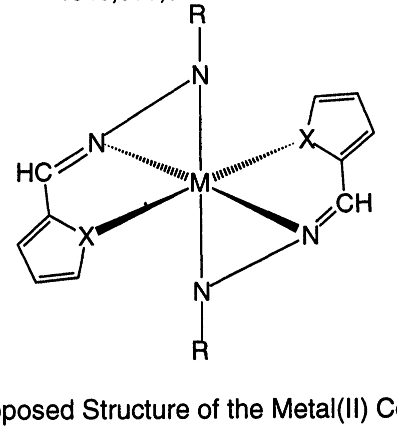

Fig. 2. Proposed Structure of the Metal(II) Complexes.

### Antibacterial Studies

The uncomplexed ligands in comparison to their metal complexes were tested for their antibacterial activity against bacterial species such as *Staphylococcus aureus, Pseudomonas aeruginosa, Klebsiella pneumonae* and *Proteus vulgarus*.The antibacterial activity was tested at concentration 30 μg / 0.01 mL in DMF by a method deviced and reported by us elsewhere[28-31].

**Table 3** Antibacterial Activity Data

| Ligands/ Complexes | Microbial Species a | b | c | d |
|---|---|---|---|---|
| $L_1$ | ++ | + | ++ | ++ |
| $L_2$ | ++ | ++ | - | ++ |
| 1 | +++ | ++ | +++ | +++ |
| 2 | +++ | ++ | +++ | +++ |
| 3 | +++ | ++ | +++ | ++ |
| 4 | ++ | +++ | ++ | +++ |
| 5 | +++ | ++ | +++ | +++ |
| 6 | ++++ | +++ | +++ | ++ |
| 7 | +++ | ++ | ++ | +++ |
| 8 | +++ | ++ | ++ | +++ |

a = *Staphylococcus aureus,* b = *Pseudomonas aeruginosa* c = *Klebsiella pneumonae,* d = *Proteus vulgarus;* Inhibition zone diameter (mm) +,**6-10**;++,**10-14**;+++,**14-18**;++++,**18-22**.

The results of these studies reproduced in table 3 show that the ligands and all their metal complexes are biologically active against one or more bacterial species. In comparison, the metal complexes have shown to be more antibacterial than the uncomplexed ligands.

### References

1. R.W.Layer, *Chem.Rev.*, **63**,489 (1963).

2. M.M.Spring, *Chem.Rev.*, **26**,297 (1940).

3. Z.H.Chohan and M.M.Farooq, *J.Chem.Soc.Pak.*, **17**,14 (1995).

4. Z.H.Chohan and H.Pervez, *Synth.React.Inorg.Met-Org.Chem.*, **23**,1061 (1993).

5. Z.H.Chohan and A.Rauf, *Synth.React.Inorg.Met-Org.Chem.*, **26**,591 (1996).

6. Z.H.Chohan and S.Kausar, *Chem.Pharm.Bull.*, **41**,951 (1993).

7. Z.H.Chohan and S.Ahmad, *Sci.Int.*, **5**,149 (1993).

8. Z.H.Chohan and S.Kausar, *Chem.Pharm.Bull.*, **40**, 2555 (1992).

9. M.F.Iskandar, S.E.Zayan, M.A.Khalifa and L.El-Sayed, *J.Inorg.Nucl.Chem.*, **36**, 556 (1974).

10. R.C.Aggarwal, N.K.Singh and R.P.Singh, *Inorg.Chem.*, **20**, 2794 (1981).

11. B.Singh, R.N.Singh and R.C.Aggarwal, *Synth.React.Inorg.Met-Org.Chem.*, **14**, 815 (1984).

12. M.F.Iskandar, L.El-Sayed, A.F.M.Hefny and S.E.Zayan, *J.Inorg.Nucl.Chem.*, **38**, 2209 (1976).

13. Z.H.Chohan and S.K.A.Sherazi, in preparation.

14. A.I.Vogel, " A Text Book of Quantitative Inorganic Analysis" , ELBS and Longman, (1973).

15. K.Nakamoto, " Infrared and Raman Spectra of Inorganic and Coordination Compounds ", J.Wiley, New York, (1978).

16. W.J.Geary, *Coord.Chem.Rev.*,**7**, 81 (1971).

17. A.M.Shallary, M.M.Moustafa and M.M.Bekheit, *J.Inorg.Nucl.Chem.*, **41**, 267 (1979).

18. A.B.P.Lever, *Inorg.Chem.*, **4**, 763 (1965).

19. B.N.Figgis and J.Lewis, *Prog.Inorg.Chem.*, **6**, 87 (1964).

20. M.D.Glick and R.L.Lintvedt, *Prog.Inorg.Chem.*, **21**, 233 (1976).

21. K.K.Barefield, D.H.Busch and S.M.Nelson, *Quart.Rev.*, **22**, 457 (1968).

22. A.D.Liehr, *J.Phys.Chem.*, **67**, 1314 (1967).

23. R.L.Carlin, " Transition Metal Chemistry", Ed. R.L.Carlin, Marcel Decker, New York, NY, Vol 1, (1965).

24. A.B.P.Lever, " Inorganic Electronic Spectroscopy", Elsevier, Amsterdam, (1968).

25. D.W.Meak, R.S.Drago and T.S.Piper, *Inorg.Chem.*, **1**, 285 (1962)..

26. B.N.Figgis and J.Lewis, "Modern Coordination Chemistry", Interscience, New York, NY (1960), Ed. T.Lewis and B.R.G.Wilkin.

27. C.Natarajan, C.D.Sheela and D.R.Athapan, *Ind.J.Chem.*, **29A**, 569 (1990).

28. Z.H.Chohan and M.A.Farooq, *Pak.J.Pharmaceut.Sci.*, **7**(, 45 (1994).

29. Z.H.Chohan and A.Rauf, *J.Inorg.Biochem.*,**46**, 41 (1992).

30. Z.H.Chohan and H.Pervez, *Pak.J.Pharmacol.*,**10**, 17 (1993).

31. Z.H.Chohan and H.Pervez, *Pak.J.Pharmaceut.Sci.*, **6**, 17 (1993).

**Received: February 7, 1997 - Accepted: March 5, 1997 - Received in revised camera-ready format: March 7, 1997**

